# Thermodynamical Analysis and Optimization of Dry Reforming and Trireforming of Greenhouse Gases: A Statistical Approach

**DOI:** 10.1021/acsomega.5c03980

**Published:** 2025-07-23

**Authors:** Jerzy Szczygieł, Karol Postawa, Katarzyna Chojnacka, Dawid Skrzypczak, Grzegorz Izydorczyk, Marek Kułażyński

**Affiliations:** † Department of Advanced Material Technologies, Faculty of Chemistry, 49567 Wrocław University of Science and Technology, Smoluchowskiego 25, Wrocław 50-372, Poland; ‡ Innovation and Implementation Company Ekomotor Ltd., Wyścigowa 1A, Wrocław 53-011, Poland

## Abstract

The paper deals with thermodynamic analysis of CH_4_ reforming with different oxidants (CO_2_, H_2_O, and O_2_) in DRM and TRM processes. Both processes producing syngas use simultaneously two components of greenhouse gases as feedstock: CO_2_ and CH_4_. Statistical methods (Response Surface Methodology, Ridge Analysis) were used to analyze the effects of temperature, pressure, and molar ratio of oxidants to methane on feedstock conversion, yield and selectivity of products, H_2_ and CO, and the H_2_/CO ratio characterizing the suitability of syngas for various syntheses. The problem of the propensity of carbon deposition in the Dry Reforming of Methane (DRM) process through the selection of operational process conditions was minimized, and in the case of the TRM process was reduced completely. The Dry Reforming of Methane (DRM) process, which is a source of synthesis gas for the subsequent synthesis of long-chain hydrocarbons (H_2_/CO = 1), is recommended to be carried out at high temperature (1273 K), low pressure (1 atm) with a molar ratio of CO_2_/CH_4_ in the feedstock of about 1. Increasing the proportion of CO_2_ in the feedstock reduces the cooking of the catalyst but at the same time reduces the hydrogen yield. Additional oxidants (O_2_, H_2_O) introduced into the system define the TRM process and enable the production of synthesis gas with a composition suitable for methanol production (H_2_/CO = 2). A positive effect on increasing the H/CO ratio is the addition of H_2_O, which intensifies the WGS reaction in the system. Both oxidants (O_2_, H_2_O) protect to some extent against catalyst cooking, but at the same time reduce hydrogen yield and CO_2_ conversion, as they are more reactive in the reaction with CH_4_.

## Graphical abstract

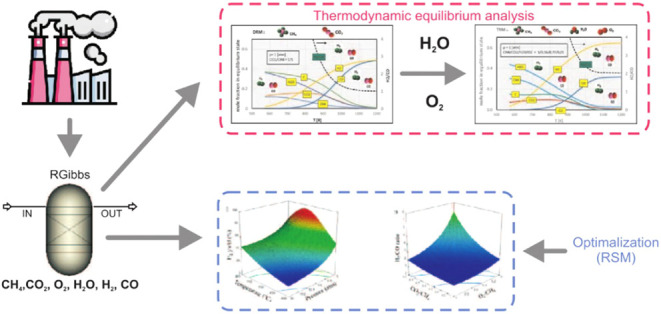

## 1 Introduction

The combustion of fossil fuels, such as coal, crude oil, and natural gas, causes a concentration of carbon dioxide in the atmosphere. At the same time, another damaging phenomenon taking place during the period of climate change that has already begun is the release of huge amounts of methane from permanently frozen geological deposits. Nonetheless, the biggest supplier of methane to the atmosphere is human activities related to agriculture, including rice cultivation and cattle breeding. Methane has significant global warming potential, and along with the independently increasing amount of CO_2_ will cause a further progressive change in the global climate.^1–3^ Both these gases are most responsible for the so-called anthropogenic greenhouse effect, which has recently been generally recognized as a primary environmental threat and a factor in the very dangerous global warming of the Earth’s climate.

In the presence of a growing problem, the reduction and utilization of already emitted greenhouse gases, such as carbon dioxide (CO_2_) and methane (CH_4_), is therefore becoming increasingly important.^4–7^ The method of CO_2_ capture and geological storage (CCS—Carbon Capture and Storage), not yet used on an industrial scale, is being promoted in the European Union as a way to reduce CO_2_ emissions from factories and power plants. Under the European Union’s project BioRECO2VER, research has been undertaken into the possibilities of converting carbon dioxide by microorganisms into compounds (e.g., isobutylene and lactates), used for the generation of valuable chemical products among others, bioplastics (e.g., tires). The UN report shows that global warming cannot be halted without parallel efforts to drastically reduce methane emissions. The pandemic and the slowdown of the economy did not limit its emissions. Under the pressure of time, even ideas are being born to capture methane from the atmosphere using the porous molecular structure of zeolites and aluminosilicate materials.

### 1.1 Chemical Utilization of Greenhouse Gases

A certain role in the greenhouse effect control has the conversion and utilization of already produced CO_2_ and CH_4_. Although the management of CO_2_ and CH_4_ represents a small scale compared to the level of emissions that occur in the energy industry, modern technologies for converting these gases into useful products are becoming increasingly important in an era of global warming. In Table 1, the reactions at the heart of these chemical technologies are presented. They are processes of CH_4_ reforming, which are carried out by using various oxidizing agents: H_2_O, CO_2_, and O_2_ are the source of synthesis gas—a mixture of H_2_ and CO—the raw material for the synthesis of many valuable products. These processes are represented by characteristic sets of chemical reactions with varying energy effects, which decide on thermodynamic determinants and privileged conditions of the course of specific processes.^8^ The methane reforming can be conducted in processes (Table 1): steam reforming (SRM),^9–19^ dry reforming (DRM),^20–23^ partial oxygenation of methane (POM),^24–28^ autothermal reforming (ATR),^29–32^ or trireforming (TRM).^28,33–38^ The molar H_2_/CO ratio in the product (synthesis gas) depends on the type of oxidant used in a given process. Steam reforming (SRM) was developed on industrial scale in the 1930s, and since then has become the primary technology for producing hydrogen and syngas. This process gives the highest molar ratio of H_2_/CO in the product, about 3. From the perspective of synthetic hydrocarbon production, it is nonoptimal for the Fischer–Tropsch process, because high H_2_/CO ratios limit carbon chain growth.^39^ High water consumption is also a problem with this technology. One of the most promising technologies, which allowed the utilization of both these undesirable greenhouse gases (CO_2_ and CH_4_) simultaneously as reactants in a single process, is the dry reforming of methane (DRM).^40,41^ Although the scale of the potential for utilization of CO_2_ and CH_4_ compared to the level of emissions from power generation is small, DRM could use natural gas deposits as feedstock, which contain large amounts of CO_2_ and are currently not economically viable for extraction. Suhartanto et al., in their work,^42^ confirmed the possibility of DRM technology application with this type of feedstock. The raw material in this process can also be biogas generated in the anaerobic digestion of organic materials, which contain CO_2_ and CH_4_ in the proportions required for the main reaction of this process. Synthesis gas obtained from the Dry Reforming of Methane (DRM) process, due to its composition (H_2_/CO ≈ 1), is suitable not only for production of long-chain hydrocarbons in FT process but also for synthesis of organic oxidized compounds (methanol, acetic acid). Another major advantage of CH_4_/CO_2_ reforming is the possibility of obtaining CO with a very high degree of purity. While in the steam reforming process, the final product (synthesis gas) contains about 2% of unreacted CH_4_, the methane concentration in the product of the “dry” reforming does not exceed 0.1%. The biggest technical challenge of DRM and an obstacle to commercialization is deactivation of catalyst caused by the formation of coke in side reactions. Many publications are devoted to researchers’ efforts to minimize the problem of DRM catalyst coking.^43^ SRM and DRM are exceptionally endothermal and require high temperatures of work in order to achieve reasonable equilibrium conversions. It leads to high operational costs. Besides, the need to operate in such harsh conditions may result in catalyst deactivation by sintering or coke deposition. Thus, for cost reduction, partial oxidation of methane (POM) is being considered as an alternative. It is a slightly exothermic reaction and the H_2_/CO ratio in the resulting synthesis gas is about 2, which makes it suitable for market competition with DRM. Coupling of SRM and POM gives an opportunity to use of the emitted heat of the POM process (exothermal) to conduct SRM (endothermal). Such an autothermal process (ATR) is energetically more efficient and the obtained H_2_/CO ratio in synthesis gas indicates its suitability for hydrogen production.^44^

**Table 1:** Main and Side CH_4_ Reforming Reactions^a^

Process	Main reaction	$\Delta H_{298}^{{}^{\circ}}$ (kJ/mol)	Reaction ID
Steam Reforming of Methane (SRM)	$CH_{4}+H_{2}O↔3H_{2}+CO$	206.2	R1
Dry Reforming of Methane (DRM)	$CH_{4}+CO_{2}↔2H_{2}+2CO$	247.0	R2
Partial Oxidation of Methane (POM)	$CH_{4}+\frac{1}{2}O_{2}↔CO+2H_{2}$	–35.0	R3
Autothermal Reforming (ATR)	$CH_{4}+H_{2}O↔3H_{2}+CO$	206.2	R4
	$CH_{4}+CO_{2}↔2H_{2}+2CO$	247.0	R5
	$CH_{4}+2O_{2}↔CO_{2}+2H_{2}O$ (MC)	–880.0	R6
Trireforming	Combination SRM, DRM, POM		
Accompanying and side reactions:			
Water Gas Shift (WGS)	$CO+H_{2}O↔H_{2}+CO_{2}$	–41.0	R7
Reverse Water Gas Shift (RWGS)	$CO_{2}+H_{2}↔CO+H_{2}O$	41.0	R8
CH_4_ decomposition (MD)	$CH_{4}↔C_{\left(s\right)}+2H_{2}$	74.9	R9
Boudouard reaction (BR)	$2CO↔C_{\left(s\right)}+CO_{2}$	–171.0	R10
Coke Gasification (−BR)	$C_{\left(s\right)}+CO_{2}↔2CO$	171.0	R11
Water Gas Reaction	$C_{\left(s\right)}+H_{2}O↔CO+H_{2}$	131.4	R12
Coke combustion	$C_{\left(s\right)}+O_{2}↔CO_{2}$	–393.7	R13
Water Gas Reaction (CW)	$C_{\left(s\right)}+2H_{2}O↔2H_{2}+CO_{2}$	90.0	R14
Sabatier reaction (reverse)	$CH_{4}+2H_{2}O↔4H_{2}+CO_{2}$	164.9	R15
CO reduction	$CO+H_{2}↔C_{\left(s\right)}+H_{2}O$	–131.4	R16

a Source: Adapted from ref 56.

A very attractive approach to syngas production has proven to be exhaust gases reforming by a reaction with methane. The process is known as trireforming (TRM) and was proposed by Song and Pan in 2004.^45^ Direct raw material for this process may be exhaust fumes from fossil-fuel-fired power plants. This exhaust usually with a composition of 12–14% CO_2_, 8–10% H_2_O, 3–5% O_2_, and 72–77% N_2_ does not have to be preseparated, as CO_2_, H_2_O, and O_2_ will be used as coreagents for the TRM process. In fact, the TRM is therefore a connection of three main reactions taking place in one reactor: steam reforming of methane (SRM), dry reforming of methane (DRM), and partial oxidation of methane (POM). The heat released from exothermal POM is utilized in the two remaining endothermic reactions, SRM and DRM, which improve the energy efficiency of the whole process. The TRM process offers additional advantages compared to the other processes mentioned above. In addition to the possibility of direct use of flue gases as raw material, the presence of H_2_O and O_2_ in the feedstock can positively influence the stability of the catalyst by inhibiting its coking, which can be a serious problem in the stand-alone Dry Reforming of Methane (DRM) process. By change of the proportions of H_2_O and O_2_ in the raw material, it is possible to influence the H_2_/CO ratio in the syngas. The H_2_/CO ratio in the TRM process may vary between 1.5 and 2.0, and it is the optimal synthesis gas composition for methanol production, as well as for other liquid fuels by Fischer–Tropsch synthesis.^37^

In recent years, there have been numerous theoretical studies based on the thermodynamic analysis of hydrogen and syngas production processes. Analysis based on Gibs’ free energy minimization method^12,46–49^ allows, bypassing the experiment, to define the equilibrium composition of the reaction mixture for given process conditions. Consequently, this method makes it possible to determine the impact of these conditions on the process efficiency and their optimization. In particular, a considerable amount of work in this area concerned methane reforming with carbon dioxide.^35,50–52^ However, the authors of these works, when analyzing the influence of various process parameters and their interactions on process efficiency, very rarely supported themselves by statistical analysis methods, e.g., Response Surface Methodology (RSM).

Comparisons between experimental and simulated data, published in the literature, are used to validate the simulated results. Experimental studies confirm the effectiveness of the Gibbs free energy minimization method and statistical methods in analyzing DRM and TRM processes.^53^ The authors of related studies^44,45,50,54,55^ have shown that experimental results were in good agreement with thermodynamic predictions.

In this paper, statistical methods were used to study the DRM and TRM reforming processes to develop the results generated earlier by a computer experiment conducted based on Aspen Plus modules and concerning the thermodynamic analysis of DRM and TRM processes.^56^ The statistical optimization methods used in this paper, unlike nonlinear programming (SQP) methods, are simple and fast to use. They do not propose as a result the coordinates of the optimal point alone but show the sensitivity of the process to changes in all process parameters simultaneously over the entire range of their variability studied.

The Response Surface Methodology technique made it possible to carry out the so-called conditional optimization, which determined the ranges of process parameter values that guarantee the simultaneous achievement of satisfactory values of several process performance indicators.^12,22,57,58^ On the other hand, the ridge analysis method allowed for determining the directions of changes in the values of process parameters and determining the local extremes of the values of process performance indicators as a function of the radius of the experiment. Based on the capabilities of the two methods, the influence of process parameters was determined; then, optimization of their values was made, and a comparison was made of the potential capabilities of each system for achieving the expected values of performance indicators.

## 2 Methodology

### 2.1 Gibbs Free Energy Minimization Method

The results of the computer experiment, which were the basis of the statistical analysis in the paper, were obtained based on the modules of the Aspen Plus, which allow the study of reaction systems in an equilibrium state.^56^ The method of Gibbs free energy minimization, implemented in the RGibbs module, gives the possibility to obtain equilibrium concentrations of components in the system without the need to specify the occurring reactions. It requires only defining the chemical species present in the system (substrates and products). The method is based on the fact that the system is thermodynamically preferred when its total Gibbs free energy expressed as a function of temperature, pressure, and concentration of the system’s components takes on a minimum value. Equilibrium conversion rates, selectivity, and yield with respect to the components of the system under study (DRM, TRM) remaining in equilibrium were defined in the following equations:

1 $$CH_{4}\;conversion\left(\%\right)=\frac{{[CH_{4}]}_{in}-{[CH_{4}]}_{out}}{{[CH_{4}]}_{in}}\times 100\%$$

2 $$CO_{2\;}conversion\left(\%\right)=\frac{{[CO_{2}]}_{in}-{[CO_{2}]}_{out}}{{[CO_{2}]}_{in}}\times 100\%$$

3 $$H_{2\;}selectivity\left(\%\right)=\frac{{[H_{2}]}_{out}}{2\times \left\{{[CH_{4}]}_{in}-{[CH_{4}]}_{out}\right\}}\times 100\%$$

4 $$\mathrm{CO}\;selectivity\left(\%\right)=\frac{{[CO]}_{out}}{\left\{{[CH_{4}]}_{in}-{[CH_{4}]}_{out}\right\}+\left\{[CO_{2}{]}_{in}-[CO_{2}{]}_{out}\right\}}\times 100\%$$

5 $$CO\;yield\left(\%\right)=\frac{{[CO]}_{out}}{{[CH_{4}]}_{in}+{[CO_{2}]}_{in}}\times 100\%$$

6 $$Carbon\;yield\left(\%\right)=\frac{{[C]}_{out}}{{[CH_{4}]}_{in}+{[CO_{2}]}_{in}}\times 100\%$$

7 $$H_{2}\;yield\left(\%\right)=\frac{{[H_{2}]}_{out}}{2\times {[CH_{4}]}_{in}}\times 100\%$$

Among these, [N]­in and [N]­out are the molar flow rates of species N (CH_4_, CO_2_, H_2_, CO, C) at the inlet and outlet of the reactor, respectively. They are further used for statistical analysis in this article.

### 2.2 Statistical Methods: Response Surface Methodology (RSM) and Ridge Analysis

The results obtained at the stage of minimization of Gibbs free energy^56^ can be treated as the results of a computer experiment obtained from the implementation of a multilevel statistical plan. The results of equilibrium conversions, equilibrium concentrations of the reforming reactants, and the degree of catalyst coking calculated in the Aspen Plus program for appropriate values of process parameters (forming the so-called factor space) were described by second-order polynomial models of the form:

8 $$Y=b_{0}+\overset{p}{\underset{i=1}{\sum }}b_{i}x_{i}+\overset{p-1}{\underset{i=1}{\sum }}\overset{p}{\underset{j=i+1}{\sum }}b_{ij}x_{i}x_{j}+\overset{p}{\underset{i=1}{\sum }}b_{ii}x_{i}^{2}$$

After adequacy tests, the models were subjected to classical statistical analysis methods. The main objective was to determine the influence and interaction of the process parameters (*x_i_*) and to quickly optimize these parameters to increase efficiency. The transformation of polynomial models to the canonical form allows for clear maps (in the system of two variables) that show the areas of optimal values of process parameters that meet the assumed criteria for several objective functions (conditional optimization). Thus, the charts will show the simultaneous influence of two process parameters on the quantities that characterize the efficiency of the process, so the interactions of these parameters at different levels will be taken into account. Hoerl’s ridge analysis optimization method was performed for the multidimensionality of the problem (number of process parameters >3) based on polynomial models. It allows the process to be analyzed in a clear and easy way to interpret two-dimensional graphs without regard to the dimensionality of the problem. This method, in contrast to other numerical methods, does not give only the coordinates of the optimal point but shows the sensitivity of the process to changes in all process parameters in the whole examined range of their variability. The method proposes different sets of process parameter values for local optima lying on the ridgeline.

## 3 Calculation Details

### 3.1 Aspen Plus—Thermodynamic Calculations

The components present in the examined systems, DRM and TRM, were defined.^56^ The systems provide for the presence of carbon as one of the components remaining in the thermodynamic equilibrium. For the calculation requirements of the program, it was assigned the status of the standard inert solid component, which does not participate in phase equilibriums. The program anticipates the presence of such a system component by assigning it the status “solids”. Other components participating in the phase equilibrium were assigned the status “conventional”. The next step involves selecting a thermodynamic method to estimate properties such as the values of constant reaction equilibriums, enthalpy, and density. The program offers here a number of various methods, depending on the sort and type of substances present in the examined system. In this study “SRK” (*Soave–Redlich–Kwong equation of state*) was adopted to be the base method, as it proves effective in most of the similar systems with a single component.

Chemical equilibria set for the proposed components in various temperature and pressure values were examined using the features and properties of the RGIBBS theoretical reactor. It allows (through the *minimization of Gibbs free energy*) to estimate equilibrium status in the system. The equilibrium results from the synergy of diversified energy properties for the individual reactions in the system.

### 3.2 Statistical Optimization

Statistical optimization relating to the defined efficiency parameters of the studied CH_4_ reforming processes (described by the polynomial in eq 8) was performed using *surf* and *contour* functions implemented in the *MATLAB* package. Isolines of different objective functions plotted in a single figure enabled conditional optimization. An appropriately prepared script in the MATLAB environment implemented the Hoerl optimization algorithm (ridge analysis).^59^ The differentiated polynomial (eq 8) for each independent variable *x_i_* and the introduction of the parameter λ­(*x*) into the system produced a system of linear equations.

$$\underset{-}{X}=-(A-\lambda \times I{)}^{-1}$$ 9

10 $$A=\left[\begin{array}{lllll} \left(2b_{11}-2b_{nn}\right) & b_{12} & b_{13} & ... & b_{1n}\\ b_{12} & \left(2b_{22}-2b_{nn}\right) & b_{23} & ... & b_{2n}\\ b_{13} & b_{23} & \left(2b_{33}-2b_{nn}\right) & ... & b_{3n}\\ . & . & . & . & .\\ b_{1n} & b_{2n} & b_{3n} & ... & 0 \end{array}\right]$$

The system repeatedly solved for assumed values of λ in a certain range and gives sets of values of *X* maximizing or minimizing values of *y* as a function of the experiment radius

$R=\sqrt{x_{1}^{2}+x_{2}^{2}+...x_{n}^{2}}$ , defined by specific optimal values of *x*_1_, *x*_2_, *x*_3_... parameters. The Hoerl method optimization algorithm was implemented in the following steps:

1. The eigenvalues of matrix *A* (eq 10) were found.
2. For values of λ lying in the intervals determined by computing the eigenvalues of matrix *A*, the dependence of the solutions of system (eq 9) on λ was determined by substituting a series of values of λ and solving system (eq 9).
3. For each set of solutions found in point 2 (*x*_1_, *x*_2_, *x*_3_,...,*x_n_*), $R=\sqrt{x_{1}^{2}+x_{2}^{2}+...x_{n}^{2}}$ and *Y* (eq 8) were calculated.
4. Based on the calculations in point 3, we plotted the local extremes of *Y*, and the optimal values of *x*_1_, *x*_2_, *x*_3_,...,*x_n_* is a function of *R*.

## 4 Results and Discussion

The thermodynamic analysis of the Dry Reforming of Methane (DRM), made with the support of the Aspen Plus package, allowed to know the equilibrium compositions of the reaction system of this process for different process conditions.^56^ Oxidants (O_2_ and H_2_O) implemented in the TRM variant of the CH_4_ reforming generate additional reactions, as a result of which a new thermodynamic equilibrium is established for specific process conditions. To determine the process conditions (including the quantitative composition of oxidants) for which the corresponding compositions of the reaction systems of DRM and TRM processes guarantee the achievement of the defined efficiencies (such as suitably high conversions and synthesis gas composition with the elimination of catalyst coking), statistical methods of processing the results were used. The analysis allows to define the suitability of each CH_4_ reforming variant in the aspect of hydrogen production and the possibility of controlling the composition of the synthesis gas obtained in terms of its usability as a raw material in other processes to produce valuable products. At the same time, the proposed process conditions should minimize the tendency to coke the catalyst, which from the point of view of mathematics reduces the problem to the so-called conditional optimization.

### 4.1 Dry and Trireforming—Computational Experiment

The algorithm implemented in the Aspen Plus software package, representing the theoretical reactor RGibbs, was used for the calculations. It provided information on the composition of the systems (representing the DRM and TRM processes) in equilibrium for different process conditions as well as the free energy (Δ*G*) of hypothetical reactions of the defined system. Based on the results of this computer experiment and statistical analysis methods, DRM and TRM operating conditions were optimized to obtain synthesis gas with the desired H_2_/CO ratio for specific F-T processes while eliminating carbon deposition side reactions, leading to catalyst deactivation.

It was assumed that DRM is represented by the following system components: CH_4_, CO_2_, CO, H_2_, H_2_O, and C, which are entangled in reactions 2, 4, 8, 9, 10, 11, 12, 14, 15, and 16 (Table 1) with different energy effects. Thermodynamic conditions of the TRM process can be analyzed assuming a reaction system in which 7 reactants—CH_4_, CO_2_, O_2_, H_2_O, H_2_, CO, and C—are involved in a series of reactions: R1, R2, R3, R6, R8, R9, R10, R12, and R13 (Table 1) representing the TRM process. The result of overlapping energy requirements coexisting in the reaction system is the equilibrium fractions of their reactants.

Our previous study^56^ shows the profiles of equilibrium concentrations of reactants in the DRM and TRM processes, respectively, carried out under analogous process conditions. The same molar ratio of oxidants to methane in both processes (for TRM: (H_2_O + CO_2_ + O_2_)/CH_4_ = 1; for DRM: CO_2_/CH_4_ = 1) allows for highlighting the differences resulting only from the replacement of some of the CO_2_ in the Dry Reforming of Methane (DRM) process by a mixture of other oxidants—H_2_O + O_2_ in the TRM process. As can be seen from the comparison, the presence of oxidants (H_2_O + O_2_) allows the CH_4_ reforming process to produce synthesis gas (H_2_ + CO) with a higher H_2_/CO ratio (1.5–2), making it useful as a feedstock in methanol and liquid fuel production processes. An additional benefit of introducing stronger oxidants (H_2_O + O_2_) is the limitation of carbon deposition on the catalyst, which in the case of the Dry Reforming of Methane (DRM) process is a serious problem. Excessive addition of oxidants: O_2_ + H_2_O + CO_2_ may, however, result in reduced productivity of hydrogen and conversion of CO_2_. In order for the process to meet expectations to the fullest extent possible, i.e., to enable high hydrogen yields with high CO_2_ conversion and maintain the desired H_2_/CO ratio, as well as to reduce or eliminate carbon deposition on the catalyst, it is necessary to optimize the operating conditions of the process. This applies to the optimization of temperature, pressure, and raw material composition represented by the proportion of the feedstock: O_2_/CH_4_, H_2_O/CH_4_, and CO_2_/CH_4_.

### 4.2 Statistical Analysis

#### 4.2.1 Response Surface Methodology

##### 4.2.1.1 Dry Reforming of Methane (DRM) Process: Modeling and Optimization

Statistical analysis of the results generated by Aspen for the thermodynamic equilibrium conditions of DRM allows the determination of areas defined simultaneously by 2 process parameters that guarantee high conversion, high hydrogen yields, and the expected value of the H_2_/CO ratio at low catalyst coking. Figure 1 (and Figure S1 in Appendix 1) shows the influence of all three process parameters (*T*, *p*, and CO_2_/CH_4_) on the conversion of both substrates of the Dry Reforming of Methane (DRM) process (CH_4_ and CO_2_). High conversion of both CH_4_ and CO_2_ is observed as expected at high temperatures and low pressures (Figure 1). This is explained by the high enthalpy value of the main reaction of DRM (R2), which proceeds with a change in the number of moles in the gas phase and hence the negative effect of pressure on the conversion of both reactants. The influence of both parameters is greater for CH_4_ conversion (Figure 1). Since the reactions involving CH_4_ have higher enthalpies than those involving CO_2_ (Table 1) the increase in temperature favors the increase in CH_4_ conversion more. In the proposed system, among the reactions proceeding with a change in mole number, there are more reactions involving CH_4_ than those involving CO_2_ (Table 1). Hence, the effect of pressure on CH_4_ conversion is more significant (Figure 1).

**Figure 1:**
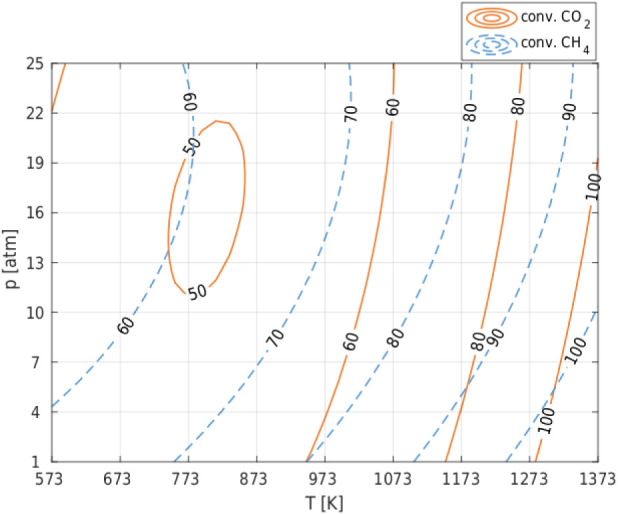
Combined effect of temperature and pressure on the conversion of CH_4_ and CO_2_ in the Dry Reforming of Methane (DRM) process for CH_4_/CO_2_ = 1 in the raw material.

The analysis of the conversion of CH_4_ and CO_2_ in the Dry Reforming of Methane (DRM) process as a function of temperature and molar CO_2_/CH_4_ ratio (Figure S1, Appendix 1) confirmed the weak reactivity and the oxidizing properties of CO_2_. The higher its proportion in the raw material mixture, the lower its conversion at each temperature of the studied range, in contrast to CH_4_ conversion, which reaches 100% at 1373 K regardless of the composition of the raw material. It also confirms the fact that the DRM feedstock is a mixture of a strong reducing agent (CH_4_) and a weak oxidizing agent (CO_2_).

Since the hydrogen yield has a simple relationship with CH_4_ conversion the effects of temperature and pressure on both parameters were the same (Figures 1 and 2). The high hydrogen yield is favored by a high temperature, a low-pressure process for which the feedstock is a mixture of CO_2_ and CH_4_ with a high predominance of methane (CO_2_/CH_4_ = 0.33) (see also Figure S2 in Appendix 1). Under these conditions, at the low coking of the catalyst, it is also possible to obtain synthesis gas of the composition corresponding to the ratio H_2_/CO = 1–2, which is favorable for the production of methanol.

**Figure 2:**
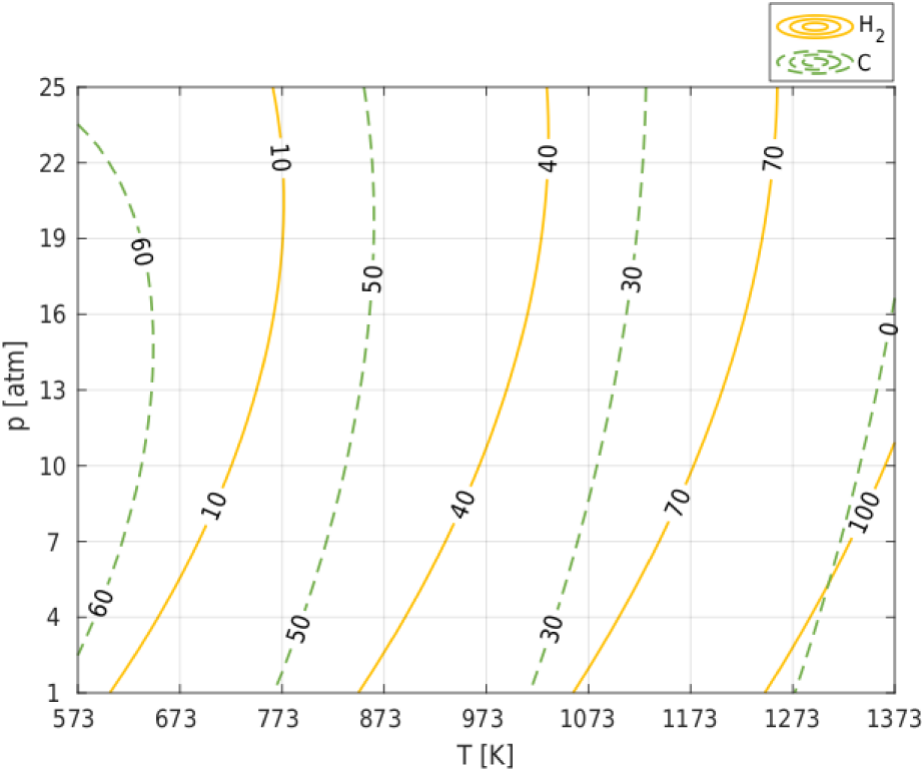
Combined effect of temperature and pressure on H_2_ efficiency and carbon deposition in the Dry Reforming of Methane (DRM) process for a CO_2_/CH_4_ ratio = 1 in the feedstock.

The process conditions maximizing hydrogen production (*T*(1373), *p*(1 atm), CO_2_/CH_4_ (0.33)) expose the catalyst to partial catalyst coking (Figure S3 in Appendix 1). To reduce this degree of coking, the proportion of CO_2_ in the feedstock would have to be increased. Then a lower hydrogen yield should be expected. This is a result of limiting the influence of the R9 reaction. In a temperature range of 573–773 K, the hydrogen yield is small (10–40%) and does not depend on the share of CO_2_ in the feedstock, while, as can be seen from Figure 3, the hydrogen yield decreases with increasing pressure.

**Figure 3:**
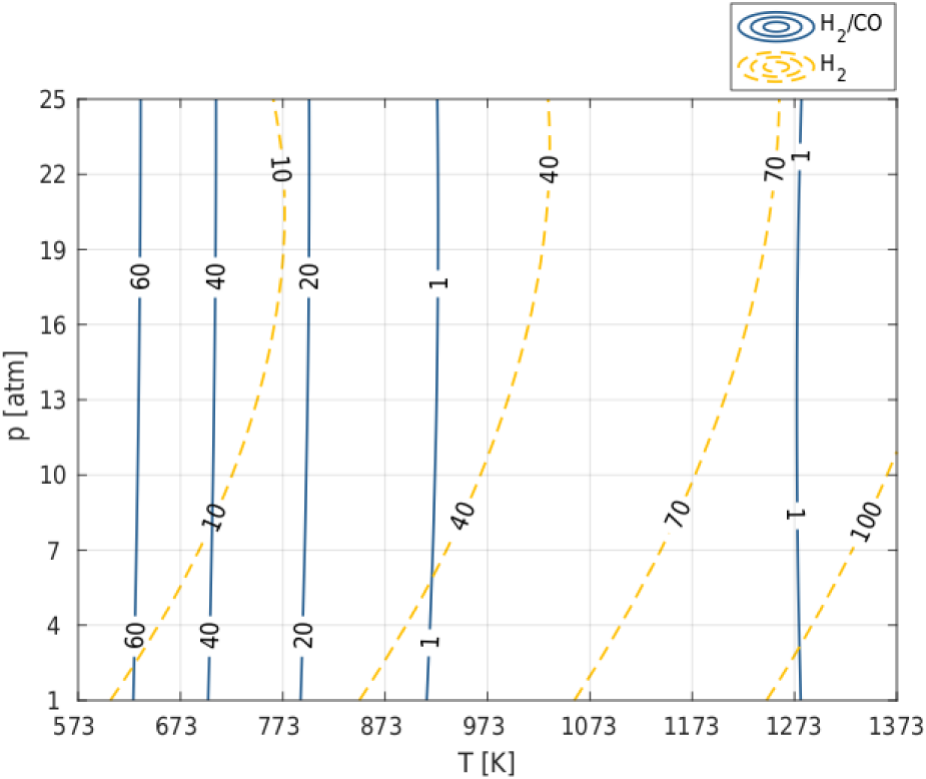
Effect of temperature and pressure on efficiency H_2_ and H_2_/CO ratio in syngas produced in the Dry Reforming of Methane (DRM) process; CO_2_/CH_4_ ratio in feedstock = 1.

H_2_/CO is one of the most important parameters of synthesis gas from the point of view of the quality of raw material for the production of various valuable products. The following reactions, which differ in their thermal effects, are responsible for their value: the CH_4_ decomposition (R9), the Boudouard reaction (R10), and the WGS reaction (R8), which proceed with H_2_ and CO. At low temperatures, the exothermic R10 reaction is more preferable to the R9 and also R8 reactions. As a result of thermodynamic conditions under these conditions, CO “disappears” from the reaction system more intensively with a moderate rate of H_2_ formation. The high value of H_2_/CO (Figure 3) is therefore due to the high value of the denominator of this expression. Thus, these are not process conditions aimed at a high hydrogen yield. At higher temperatures, due to the reverse R10 reaction and the RWGS reaction (R8), hydrogen is consumed while CO is produced, which results in a low value of H_2_/CO (Figure 3). In the temperature range where H_2_/CO < 3 the hydrogen yield increases from 40 to 80 as the proportion of the R8 (RWGS) reaction consuming hydrogen decreases.

Carbon monoxide CO is known as an industrial gas that has many industrial applications in various chemical processes such as the Fischer–Tropsch process to produce hydrocarbons. From the analysis (see Figure S4 in Appendix 1), it can be seen that in DRM, its yield increases with temperature (inverse R10 reaction), also affecting the property of the synthesis gas.

The analysis of Figures 1 and 3 (and Figures S1–S4 in Appendix 1), which are the result of response surface modeling, leads to the conclusion that to obtain simultaneously:

(1) maximum of hydrogen production,
(2) maximum conversion of methane with the minimum amount of coke deposited, and
(3) optimum ratio H_2_/CO = 1 in the synthesis gas for DRM.

DRM should be carried out at high temperature (1273 K) and low pressure (1 atm) at a CO_2_/CH_4_ molar ratio in the feedstock of about 1.

##### 4.2.1.2 Trireforming of Methane (TRM) Process: Effects of Multiple Factors and Optimization

Figures 4 and 5 (see also Figures S5–S8 in Appendix 1) focus on the interaction effect of feed ratios such as O_2_/CH_4_, H_2_O/CH_4_, and CO_2_/CH_4_ on the process efficiency criteria.

**Figure 4:**
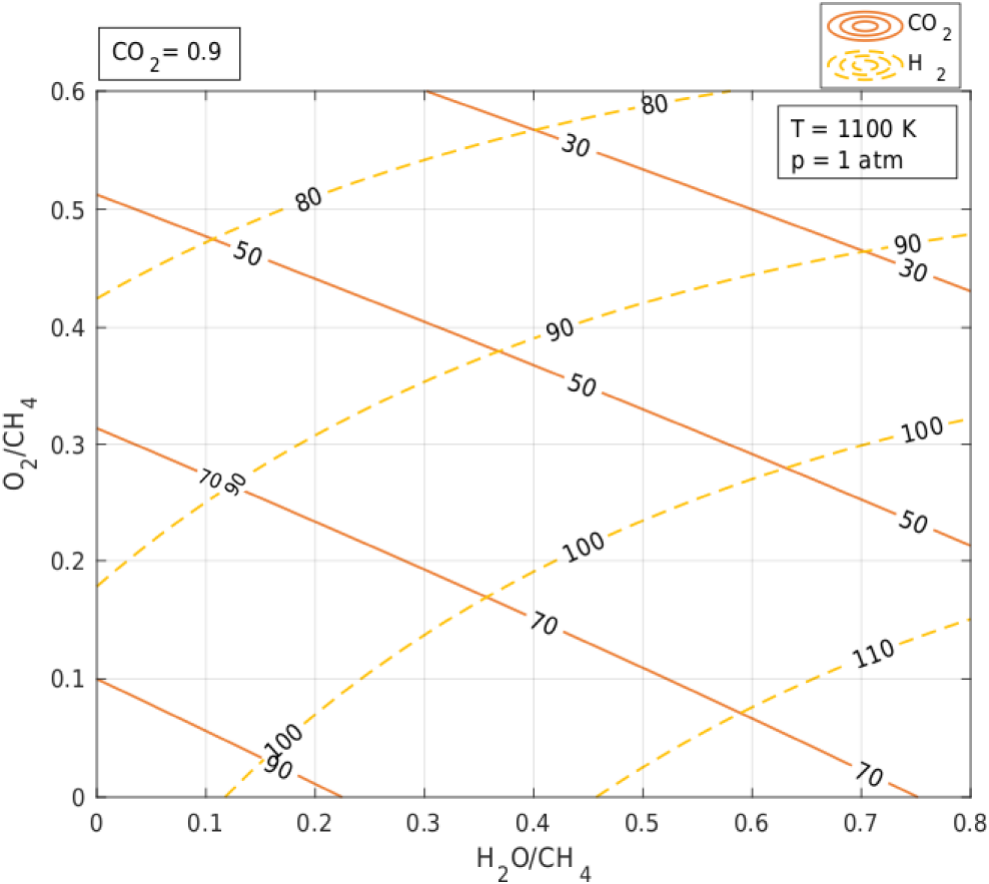
Combined effect of oxygenates (molar H_2_O/CH_4_ and O_2_/CH_4_ ratios) in the feedstock of the TRM process on conversion CO_2_ and H_2_ yield; CO_2_/CH_4_ ratio = 0.9, *T* = 1100 K, *p* = 1 atm.

**Figure 5:**
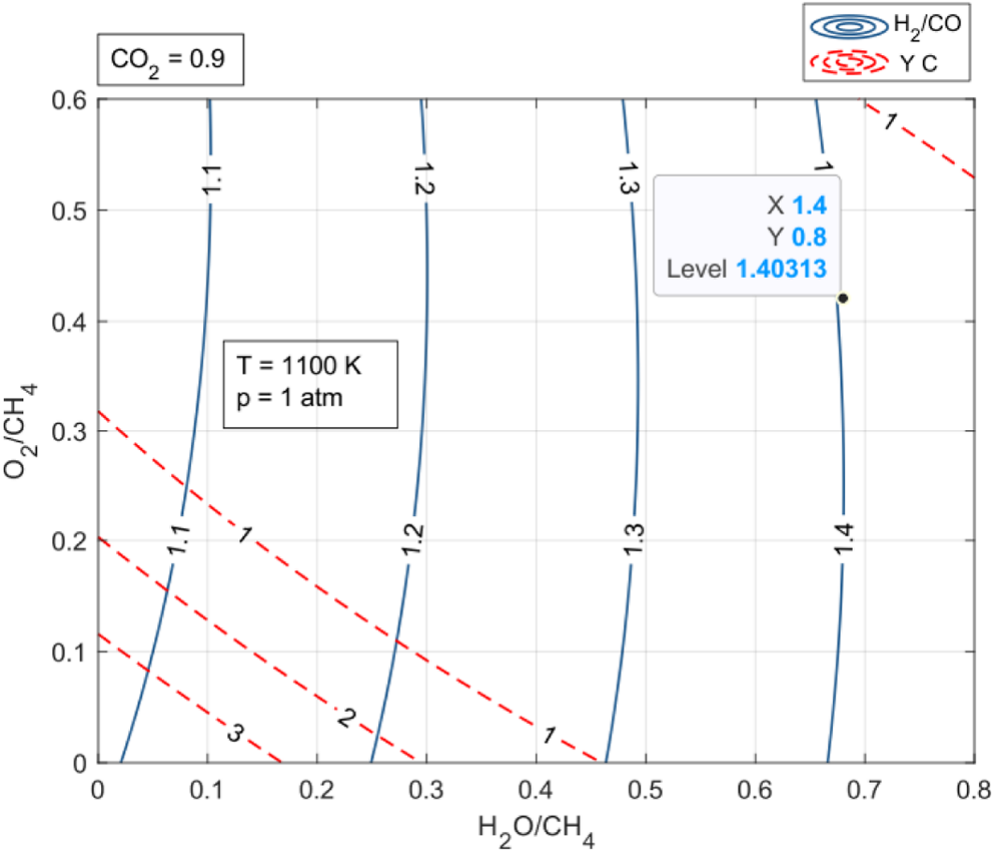
Combined effect of oxygenates (molar H_2_O/CH_4_ and O_2_/CH_4_ ratios) in the feedstock of the TRM process on carbon deposition and H_2_/CO ratio in syngas; CO_2_/CH_4_ ratio = 0.9, *T* = 1100 K, *p* = 1 atm.

Figure 4 (see also Figures S5 and S6 in Appendix 1) shows CO_2_ conversion and hydrogen yield, and Figure 5 (see also Figures S7 and S8 in Appendix 1) shows the H_2_/CO ratio and carbon formation with respect to the combined effect of the O_2_/CH_4_ and H_2_O/CH_4_ ratios for different CO_2_/CH_4_ ratios. The isolines were plotted based on the results of the (Aspen) equilibrium composition in the system representing TRM for 1100 K and 1 atm and CO_2_/CH_2_ = 0.9. The choice of high temperature and low pressure justifies the endothermic balance of TRM process reactions, which mostly proceed with increased volume (Δ*H* > 0; Chatelier’s rule).^55^ On the other hand, the high CO_2_ content of the feedstock protects from catalyst coking at a low cost of hydrogen yield (Figures S5–S8, Appendix 1).

Figure 4 (see also Figures S5 and S6 in Appendix 1) shows that a higher proportion of O_2_ in the system results in a lower hydrogen yield and lower CO_2_ conversion (at each level of the amount of CO_2_ in the feedstock), which can be explained by the increased tendency of the system components to oxidize to CO_2_ and H_2_O instead of forming hydrogen and CO. Oxygen as an additional oxidant that appears in the system under specific conditions of temperature and pressure generates new reactions that result in a new equilibrium by changing the proportions of the other TRM reactants in the system. Oxygen as a more reactive competes with CO_2_ in the reaction with CH_4_ (R2). The CH_4_ combustion reaction (R6) has an advantage over the dry reforming reaction (R2). As a result, the conversion of CO_2_ in the R2 reaction decreases. The yield of hydrogen, which is the product of the R2 reaction, also decreases because of this. Instead of CO and H_2_ from the R2 reaction, we have CO_2_ and H_2_O in the R6 reaction.

As can be seen from the same figures, the addition of H_2_O to the system also decreases the CO_2_ conversion, although as the slopes of the isolines indicate the effect of H_2_O is smaller. The explanation is that H_2_O is more reactive than CO_2_ but less reactive than O_2_ in its reaction with CH_4_ (R2). The conversion of CO_2_ by H_2_O addition also decreases (although to a lesser extent at higher temperatures) the level of the WGS reaction (R7), whose product is CO_2_. The observed increase in the proportion of hydrogen with the addition of H_2_O in the maps can be explained by the more intense course of the SRM reaction (R1) and the partial displacement of CO_2_ by H_2_O in the R2 reaction. Besides, at higher temperatures, the decomposition of methane (R9) takes place, the product of which is hydrogen and carbon, which in the endothermic reaction, WGR (R16) with water, forms the synthesis gas components H_2_ and CO.

Figure 5 (see also Figures S7 and S8 in Appendix 1) shows the variation of the H_2_/CO ratio and carbon yield in the system as a function of the O_2_/CH_4_ and H_2_O/CH_4_ (for different CO_2_/CH_4_ levels). The effect of the O_2_ content in the system on the H_2_/CO ratio, which characterizes the suitability of the synthesis gas for different processes, turns out to be negligible at different levels of the CO_2_ content in the system. On the other hand, the effect of H_2_O on the value of the H_2_/CO ratio is noticeable and positive. The addition of water intensifying the WGS reaction (R7) in which CO is the feedstock and H_2_ is the product increases the H_2_/CO ratio. An important advantage of the presence of additional amounts of oxidants, O_2_, H_2_O, and CO_2_, in the TRM process is to minimize the problem of coking and catalyst poisoning, which comes from side reactions: methane decomposition (R9) and the Boudouard reaction (R10). Additional reactants—oxidants: O_2_, H_2_O, and CO_2_—under certain conditions can take part in reactions (R11, R16, R13) by consuming carbon, thereby freeing the process from catalyst coking. As can be seen from Figure 5 (see also Figures S7 and S8 in Appendix 1), at each CO_2_/CH_4_ level in the presence of O_2_ and H_2_O, coke-free areas (determined by the equilibrium compositions of the TRM system) can be distinguished, with the inclusion of O_2_ having a greater contribution to nullifying coke deposition on the catalyst.

By comparing Figures 4 and 5 (as well as Figures S5–S8 in Appendix 1) we can evaluate the influence of the third oxidant, CO_2_, on the investigated process efficiency parameters. The individual effect of the (CO_2_/CH_4_) contribution on the CO_2_ conversion and hydrogen contribution is qualitatively similar to that of the other oxidants: O_2_ and H_2_O. It decreases CO_2_ conversion and hydrogen yield. However, it appears that at a low CO_2_ contribution, the effect of the contribution of the other oxidants is greater than for higher CO_2_ contribution.

#### 4.2.2 Ridge Analysis: Optimization

##### 4.2.2.1 Dry Reforming of Methane (DRM) Process—Optimization

The Hoerl method applied to selected optimized quantities of DRM (CH_4_ conversion, CO_2_ conversion, H_2_ yield, H_2_/CO ratio) allows for monitoring the changes in the values of the process control parameters (along the experiment radius, *R*) for its optimization. By implementation of the algorithm (described in Section 3.2), it is possible to find local maxima or minima (along with the radius *R*) of the optimized quantities and the corresponding values of the process control parameters. Thus, it is possible to follow the path of the fastest increase or decrease in the value of the dependent variable as a function of the control quantities. Graphs showing the course of the line of the fastest increase or decrease in the conversion of CH_4_, CO_2_, H_2_ yield, and parameter H_2_/CO as a function of the radius of the experiment are shown in Figure 6b (see also Figures S9b, S10b, and S11b). The limiting value of the radius of the sphere *R* (defined by the formula in Section 3.2) in the three-dimensional space for which reliable information can be obtained is assumed to be equal to 3. Figure 6a (see also Appendix 1, Figures S9a,S10a, andS11a) is useful for the determination of the ranges of values of λ for which the radius of the sphere does not exceed the adopted value, which ensures that the implemented algorithm will limit the search for locally optimal process parameters to the adopted ranges of variables.

**Figure 6:**
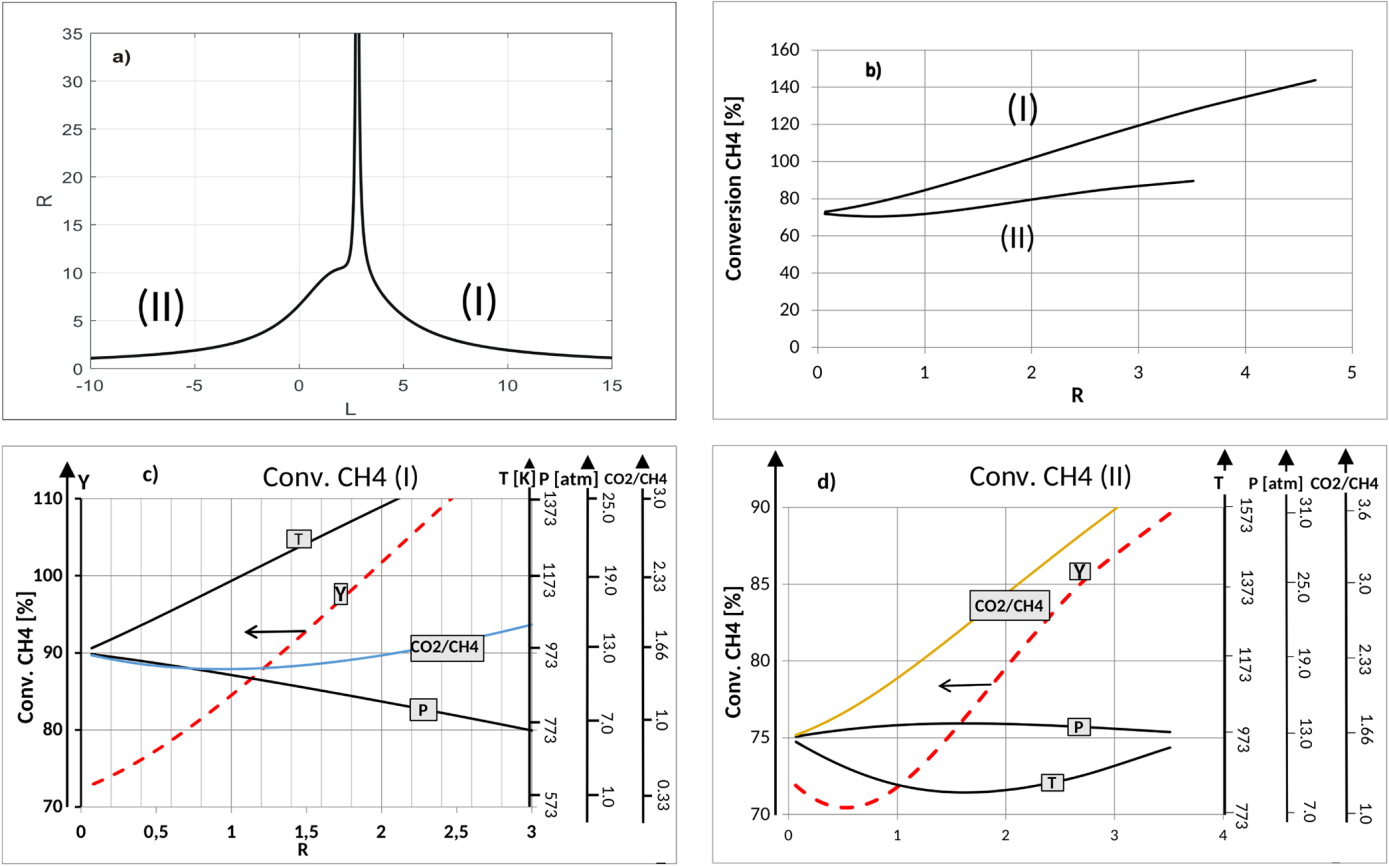
Ridge analysis for conversion CH_4_ in the Dry Reforming of Methane (DRM) process: a) Lambda versus *R*, b) local optimum responses (conversion CH_4_, cases I and II) versus *R*, c) maximum ridge coordinates for case I, d) maximum ridge coordinates for case II.

The implementation of the algorithm leads to the determination of the dependence of the solutions of the system of eqs (12) on the value of λ. The tabulated values of these solutions—*T*(λ), *p*(λ), CO_2_/CH_4_ (λ), *R*(λ), and *Y* (λ)—from the selected (based on Figure 6a as well as in Appendix 1, Figures S9a, S10a, and S11a) range of λ were used for further analysis and plotting.

Figure 6c,d are two possible options that indicate the paths to achieve the maximum CH_4_ conversions, represented by the two ridge lines I and II from Figure 6b. In the most general terms, these graphs provide us with a range of locally optimal CH_4_ conversion values (left axis) along with the corresponding process parameter values that locally provide them with the maximum value (right axis). Out of these many possibilities distributed along the “ridge”, the best one is determined by the process possibilities. Analysis of Option II suggests that high, though lower, conversion values can be obtained by realizing optionally a different trend of changes in process parameter values, as shown in Figure 6d. The choice of an option depends on the process capability of realizing these requirements.

The fastest increase in CH_4_ conversion represented by ridgeline (I) (Figure 6b) can be obtained by increasing the temperature, decreasing the pressure, and keeping the feedstock at a constant molar ratio (Figure 6c). The maximum value of the conversion is achieved at the edge of the experimental area (*R* = 2), for *T* = 1373 K, decreasing pressure and a CO_2_/CH_4_ ratio of about 1.5. Analysis of the second option indicates that (Figure 6d) 80% of CH_4_ conversion can be achieved with a lowered temperature (about 873 K) and an increased CO_2_/CH_4_ molar ratio in the feedstock (3.0). The fastest increase in CO_2_ conversion is stimulated by the same parameter changes (along the radius) of the process as in the case of CH_4_ conversion (Figure S9c in Appendix 1). The analysis of the second CO_2_ conversion ridgeline (II) (Figure S9b in Appendix 1) revealed that the fastest increase in CO_2_ conversion (although lower) can be achieved by decreasing the proportion of CO_2_ in the feedstock while keeping the temperature and pressure constant (Figure S9d in Appendix 1). 70% of the CO_2_ conversion can be achieved at *T* = about 973 K, elevated pressure (about 13 atm), and a CO_2_/CH_4_ ratio of about 0.33. If the goal is to maximize H_2_ efficiency, the analysis should be based on the ridgeline labeled (I) (Appendix 1, Figure S10b). The course of the ridgeline (in Appendix 1, Figure S10c) indicates that the hydrogen yield increases under conditions that maximize the conversion of CH_4_ as well as CO_2_, i.e., with increasing temperature and decreasing pressure while keeping the CO_2_/CH_4_ value at 1. This confirms the previous conclusions based on the analysis of the thermodynamics of the reaction and the statistical analysis performed based on the plotted isolines. The analysis of the *R*(λ) function (Figure S11a in Appendix 1) for the parameter characterizing the synthesis gas (H_2_/CO) indicates the noteworthy existence of two ridgelines because of their location inside (curve I) or on the border (curve II) of the experimental area. The process conditions allowing high values of this parameter may be interesting from a hydrogen production point of view. On the other hand, low values show the usefulness of syngas as a raw material for making valuable products. The analysis of both ridgelines shows that high values of H_2_/CO can be obtained by decreasing the process temperature while increasing the proportion of CO_2_ in the feedstock (Figure S11c in Appendix 1) or vice versa (Figure S11d in Appendix 1). If the goal is to obtain syngas with low H_2_/CO values, the analysis based on ridgeline I is more reliable because it completely runs inside the experimental plan area (*R*= 1:3).

##### 4.2.2.2 Trireforming of Methane (TRM) Process—Optimization

The results of equilibrium CO_2_ conversions, hydrogen yields, carbon contents, and H_2_/CO values calculated from equilibrium synthesis gas compositions were statistically analyzed to optimize the oxidant content of the reforming feedstock.

Hoerl’s ridgeline method allows analysis of the influence of all 5 parameters simultaneously on the determined/selected criteria.

Figure 7 shows the path of the fastest decrease in carbon content as a function of temperature, pressure, and the content of three oxidants (O_2_/CH_4_, CO_2_/CH_4_, and H_2_O/CH_4_) in the raw material. Since the graph covers the area of carbon content decline with negative numerical values, the analysis of the influence of these 5 factors can only be qualitative.

**Figure 7:**
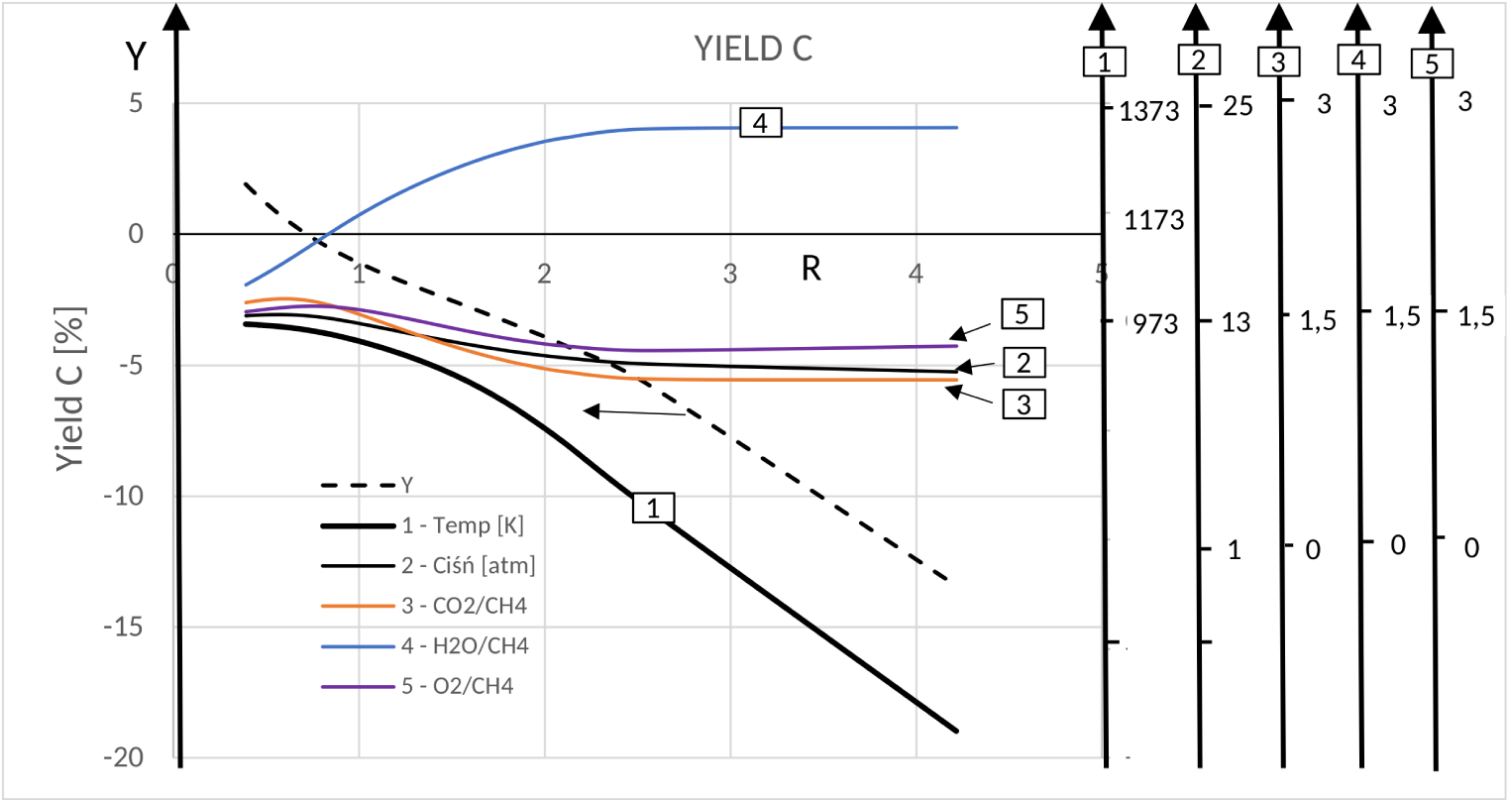
Ridge analysis: optimum ridge coordinates versus *R* for carbon deposition in the TRM process.

It can be seen that the most significant process parameters determining the carbon content of the system are the temperature and pressure. This is confirmed by the thermal effects and pressure dependence of reactions R9, R10, R11, R12, and R16 considered in the analysis, which determine the level of coking in the system. The path (ridgeline) guaranteeing the consolidation of the trend of decreasing carbon content is favored by decreasing the pressure at high temperatures and maintaining a certain level of oxidants—CO_2_, H_2_O, and O_2_ (Figure 7). The carbon that appears in the system at lower temperatures, mainly due to CH_4_ decomposition (R9), CO dissociation (R10—BR), and CO reduction (R16) disappears at higher temperatures and low pressures due to the reaction (R11). O_2_ is the first of the oxidants to be consumed regardless of the temperature with a 100% conversion. Its role in maintaining a minimum level of carbon in the system is the greatest. Thanks to its presence, it is not necessary to exceed the relatively high level of temperature for the reduction of carbon in the system as that could be harmful to the catalyst.

The local maxima of hydrogen yield and CO_2_ conversion as a function of the experimental radius increase under the condition of simultaneous changes in the values of five independent process parameters according to the trends shown in Appendix 1 (Figures S12 and S13, respectively). According to the conclusions derived from the thermodynamic analysis of the system, the increase in hydrogen yield and CO_2_ conversion is favored by increasing the temperature and decreasing the pressure. On the other hand, the effect of oxidant contribution is different. Keeping the low O_2_/CH_4_ value favors the CO_2_ conversion and hydrogen yield, with a more pronounced effect of the O_2_ (O_2_/CH_4_) proportion being seen in relation to the hydrogen yield.

## 5 Conclusions

The analysis of the results of the composition of the reaction systems under thermodynamic equilibrium conditions, carried out in this work using statistical methods, allowed to determine the process conditions and the initial composition of raw materials including the ratio of oxidants (O_2_, H_2_O, CO_2_) to CH_4_ for obtaining, potentially, the most favorable efficiencies of the DRM and TRM processes.

In the case of the Dry Reforming of Methane (DRM) process, which is a source of synthesis gas for the subsequent synthesis of long-chain hydrocarbons (H_2_/CO = 1), the process is recommended to be carried out at high temperature (1273 K), low pressure (1 atm) with a molar ratio of CO_2_/CH_4_ in the feedstock of about 1. The reduction of the CO_2_ share in the raw material admittedly reduces the coking of the catalyst, but simultaneously lower hydrogen yields are to be expected. Theoretically, it is possible to obtain in the DRM process a synthesis gas suitable for methanol production (H_2_/CO = 2) and even more so for the production of alkanes (C_1_–C_5_) demanding raw material with characteristics H_2_CO ≥ 2, but the conditions that guarantee this are not satisfactory from the point of view of coke deposition on the catalyst. On the other hand, synthesis gas with the characteristic H_2_/CO ≤ 1 can be produced without carbon release under many alternative sets of process conditions.

Additional oxidants (O_2_, H_2_O) introduced into the system by initiating new reactions slightly change its characteristics and energy requirements defining the TRM process, which allows the production of synthesis gas of a composition suitable for the production of methanol (H_2_/CO = 2). A noticeable and positive impact on the increase of H_2_/CO ratio has especially been the addition of H_2_O, which intensifies the WGS reaction in the system. Additional oxidants (O_2_, H_2_O) protect to some extent against catalyst coking, but at the same time reduce hydrogen yield and CO_2_ conversion, as they are more reactive in the reaction with CH_4_.

The second statistical technique—Hoerl’s method allowed identification of the sensitivity of the studied processes to changes in all process parameters simultaneously and the determination of the direction of changes in their values for optimization.

Generally, in the case of CH_4_ reforming, to achieve the defined goals, high temperature and low pressure are necessary. For the TRM process, by the addition of oxidants H_2_O and O_2_ one can count on lowering the temperature at which coke is formed and on the desired composition of synthesis gas.

The conclusions formulated are based on the statistical methods used in the work, which by their nature may leave a certain margin of lower reliability. Therefore, in order to strengthen them and make them credible, in many cases, they were confirmed by thermodynamic analysis using data on the thermal effects of the individual reactions that make up the DRM and TRM processes (Table 1). Such analysis clarifies the behavior of the system under changing conditions and lends credibility to the conclusions.

The optimal values of the process parameters of the two processes, DRM and TRM, proposed in this work on the basis of statistical analysis (RSM, ridge analysis) are consistent with the results presented in our previous work^56^ and the results of other works^22,60^ and are also in agreement with the results verified by experiment.^44,45,50,54,55^

## Supplementary Material



## Data Availability

The data underlying this study are available in the published article and its Supporting Information.

## Notes

The data supporting the findings of this study are available within the digital version of the article. Supporting Information includes additional contour plots showing the effect of tested pairs on the optimized parameters. The second part of Supporting Information includes figures visualizing the results of the Ridge analysis (PDF)

## Abbreviations list:

ATR: Autothermal Reforming
-BR: Coke Gasification
BR: Boudouard reaction
CW: Water Gas Reaction
DRM: Dry Methane Reforming
MD: CH_4_ decomposition
POM: Partial Oxidation
RSM: Response Surface Methodology
RWGS: Reverse Water Gas Shift
SQP: Sequential Quadratic Programming
SRM: Steam Methane Reforming
TRM: Trireforming of Methane
WGS: Water Gas Shift

## Symbols list:

*b*: linear coefficients of equation: *b*_1_, *b*_2_, *b*_3_... *b*_n_
*I*: unitary matrix of size *n* × *n*
*n*: number of independent variables
*P*: pressure
*p*: number of independent variables
*R*: experiment radius
*T*: temperature
*x_i_*: independent variables (*i* = 1 ÷ *p*)
*X*: vector of independent variables determined by λ
*Y*: dependent variables
λ: Lagrange multiplier
