# Supplementary material for: Thermodynamical Analysis and Optimization of Dry Reforming and Trireforming of Greenhouse Gases: A Statistical Approach
Source: ACS Omega. 2025 Jul 23;10(30):33536–47. doi: 10.1021/acsomega.5c03980 (PMC12332636; doi:10.1021/acsomega.5c03980)
Supplement: Supplementary file 1 [file ao5c03980_si_001.pdf]

Thermodynamical analysis and optimization of Dry Reforming and Tri Reforming of greenhouse gases, a statistical approach

Jerzy Szczygieł[b], Karol Postawa[a*], Katarzyna Chojnacka[a], Dawid Skrzypczak[a], Grzegorz Izydorczyk[a], Marek Kułażyński[b]

[a] Department of Advanced Material Technologies, Faculty of Chemistry, Wrocław University of Science and Technology, Smoluchowskiego 25, 50-372 Wrocław, Poland

[b] Innovation and Implementation Company Ekomotor Ltd., Wyścigowa 1A, 53-011 Wrocław, Poland

[*]Corresponding author e-mail: karol.postawa@pwr.edu.pl

Abstract

The paper deals with thermodynamic analysis of CH_4 reforming with different oxidants (CO_2, H_2O, O_2) in DRM and TRM processes. Both processes producing syngas use simultaneously two components of greenhouse gases as feedstock: CO_2 and CH_4. Statistical methods (response Surface methodology, ridge analysis) were used to analyze the effects of temperature, pressure and molar ratio of oxidants to methane on feedstock conversion, yield and selectivity of products - H_2 and CO, and H_2/CO ratio characterizing the suitability of syngas for various syntheses. The problem of the propensity of carbon deposition in the Dry Reforming of Methane (DRM) process through the selection of operational process conditions was minimized, and in the case of the TRM process was reduced completely. The Dry Reforming of Methane (DRM) process, which is a source of synthesis gas for the subsequent synthesis of long-chain hydrocarbons (H_2/CO = 1) is recommended to be carried out at high temperature (1273 K), low pressure (1 atm) with a molar ratio of CO_2/CH_4 in the feedstock of about 1. Increasing the proportion of CO2 in the feedstock reduces the cooking of the catalyst, but at the same time reduces the hydrogen yield. Additional oxidants (O_2, H_2O) introduced into the system define the TRM process, and enable the production of synthesis gas with a composition suitable for methanol production (H_2/CO = 2). A positive effect on increasing the H2/CO ratio is the addition of H_2O, which intensifies the WGS reaction in the system. Both oxidants (O_2, H_2O) protect to some extent against catalyst cooking, but at the same time reduce hydrogen yield and CO_2 conversion, as they are more reactive in reaction with CH_4.

Keywords

Greenhouse gases; Methane reforming; TRI – reforming; Thermodynamic analysis; Response Surface Methodology; Optimization

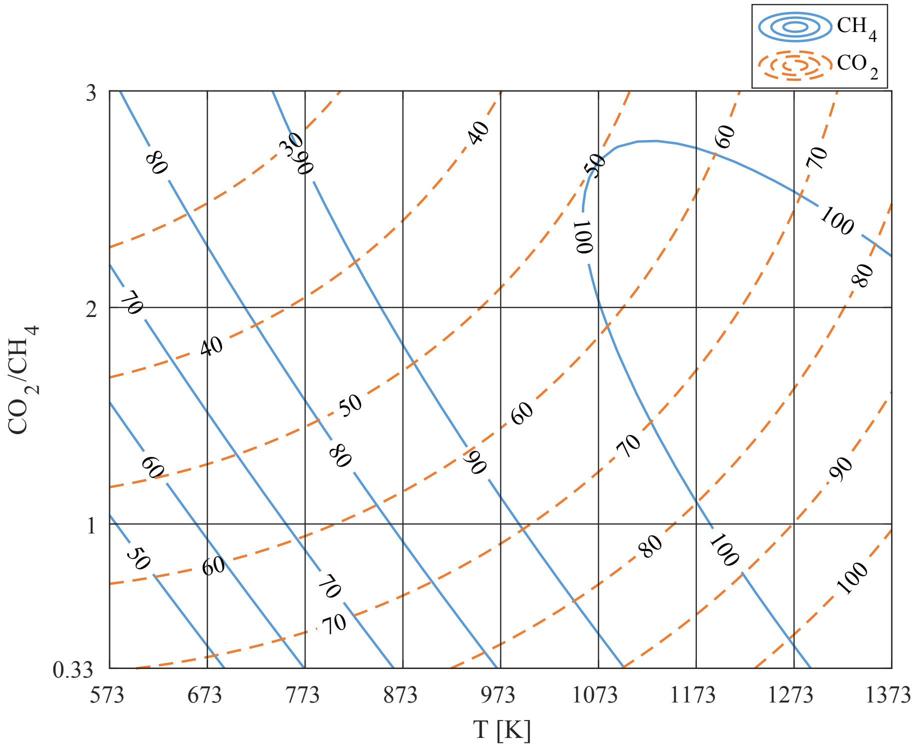

Figure S1. Conversion of CH4 and CO2 in DRM process as a function of temperature and molar CO2/CH4 ratio in raw material; p=1 atm

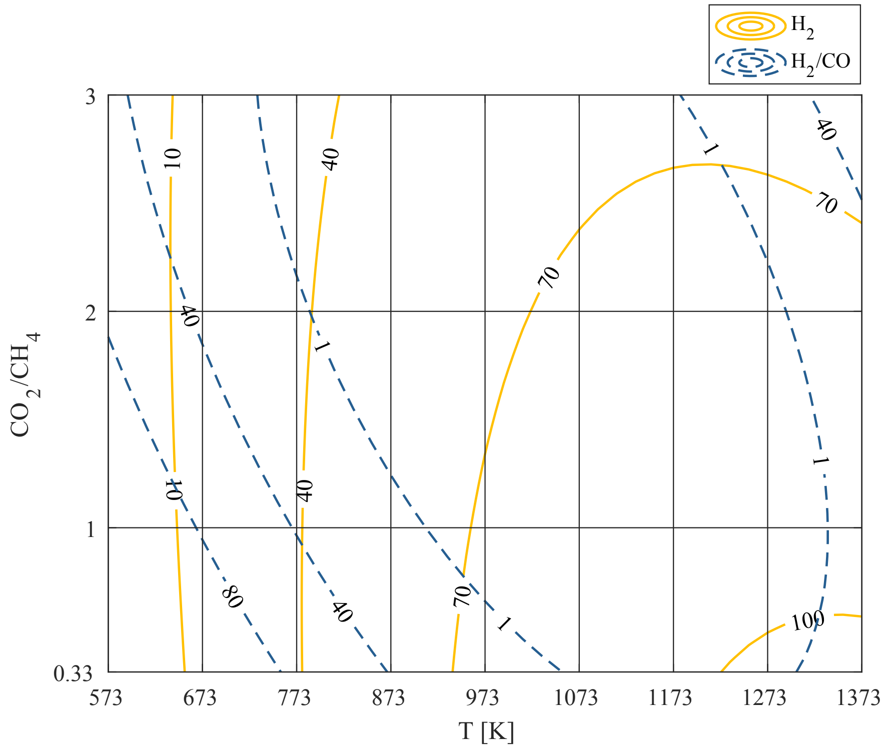

Figure S2. Effect of temperature and molar CO2/CH4 ratio in the feedstock of DRM process on efficiency H2 and H2/CO ratio in syngas; p = 1 atm

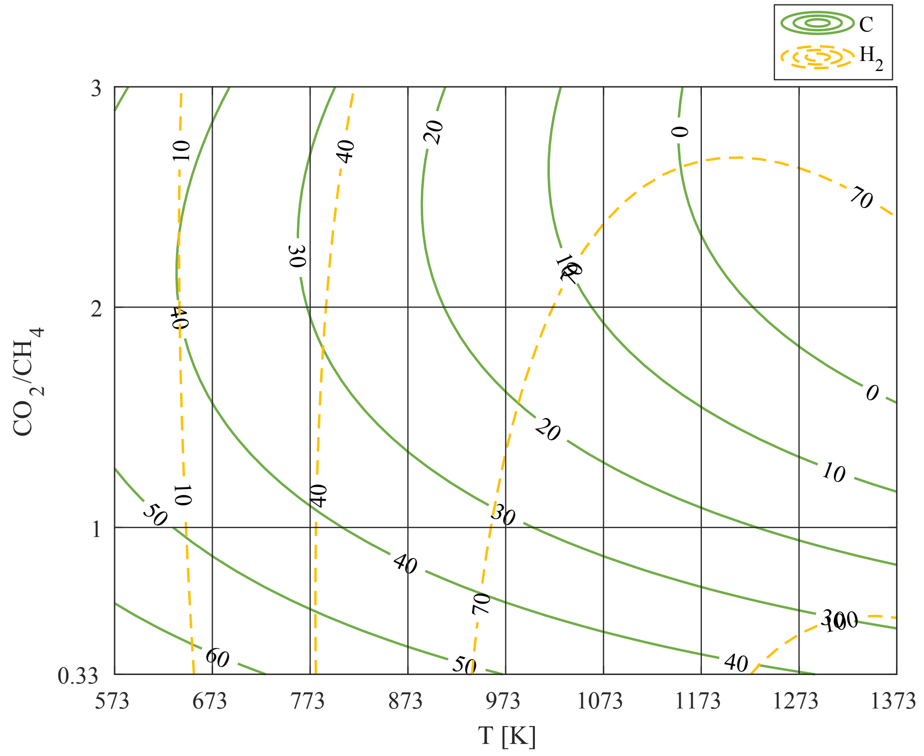

Figure S3. Combined effect of temperature and molar CO2/CH4 ratio in the feedstock of DRM process on H2 efficiency and carbon deposition; p=1 atm

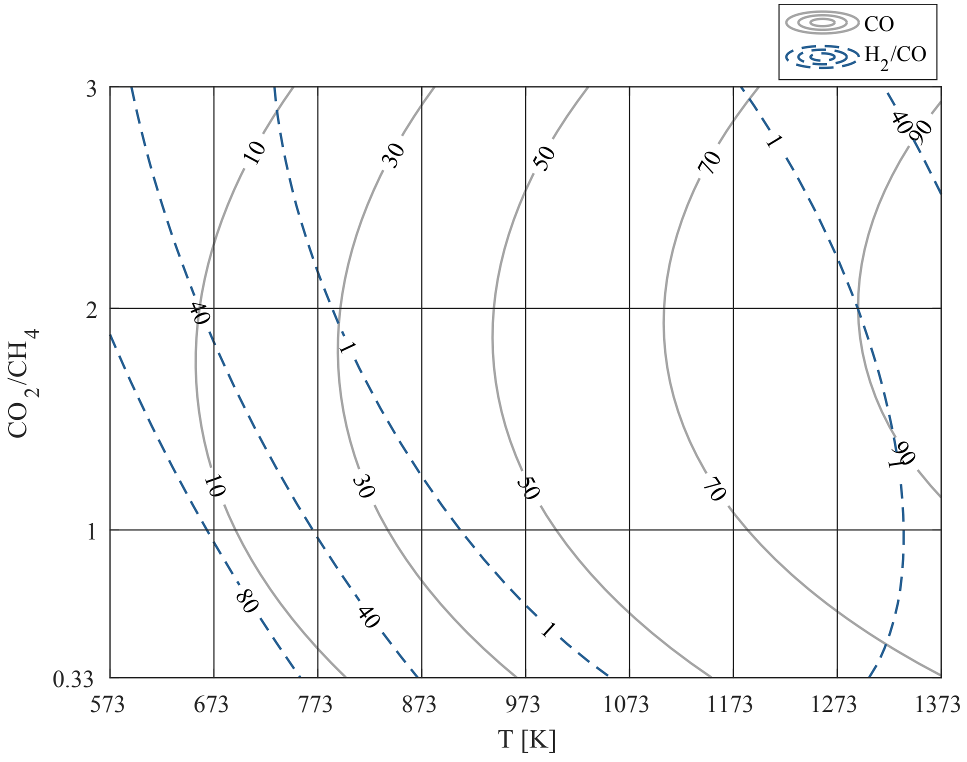

Figure S4. Effect of temperature and molar CO2/CH4 ratio in the feedstock of DRM process on CO efficiency and H2/CO ratio in syngas; p=1 atm

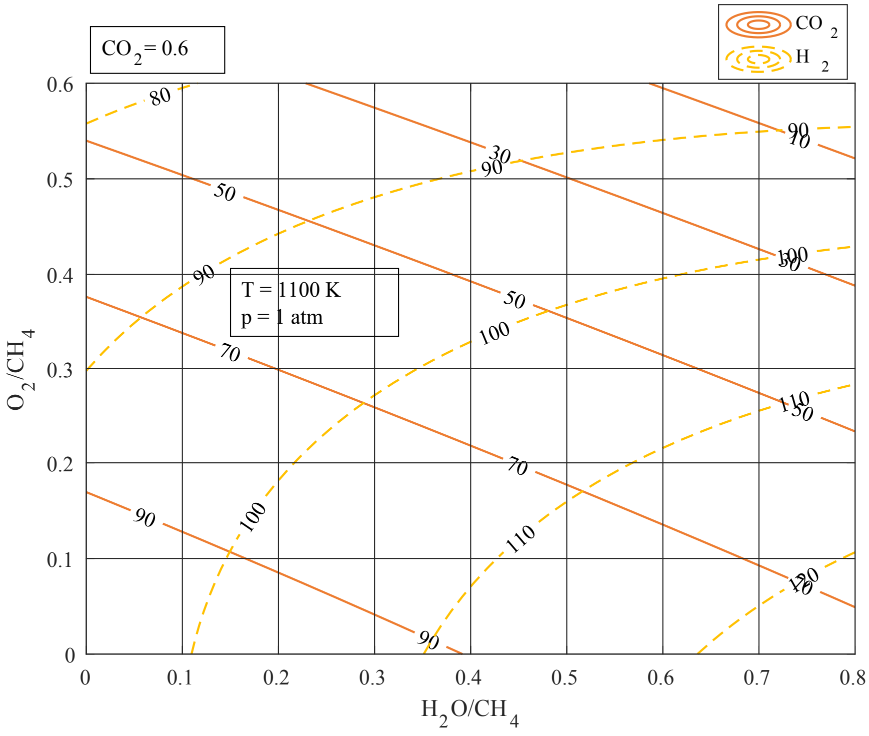

Figure S5. Combined effect of oxygenates (molar H2O/CH4 and O2/CH4 ratio) in the feedstock of TRM process on conversion CO2 and H2 yield; CO2/CH4 ratio=0,6, T=1100 K, p=1 atm

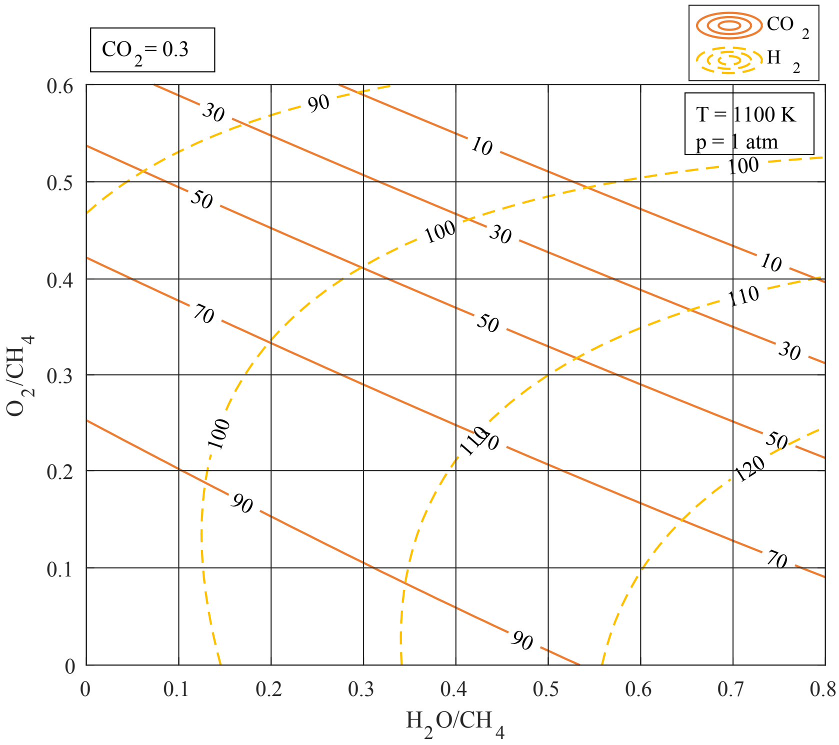

Figure S6. Combined effect of oxygenates (molar H2O/CH4 and O2/CH4 ratio) in the feedstock of TRM process on conversion CO2 and H2 yield; CO2/CH4 ratio=0,3, T=1100 K, p=1 atm

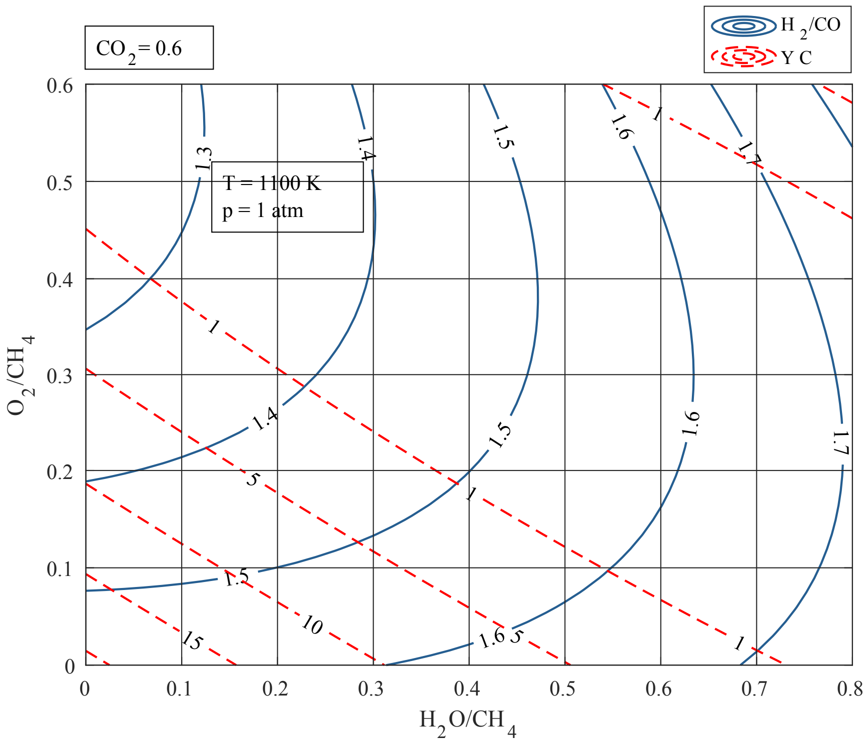

Figure S7. Combined effect of oxygenates (molar H2O/CH4 and O2/CH4 ratio) in the feedstock of TRM process on carbon deposition and H2/CO ratio in syngas; CO2/CH4 ratio=0.6, T=1100 K, p=1 atm

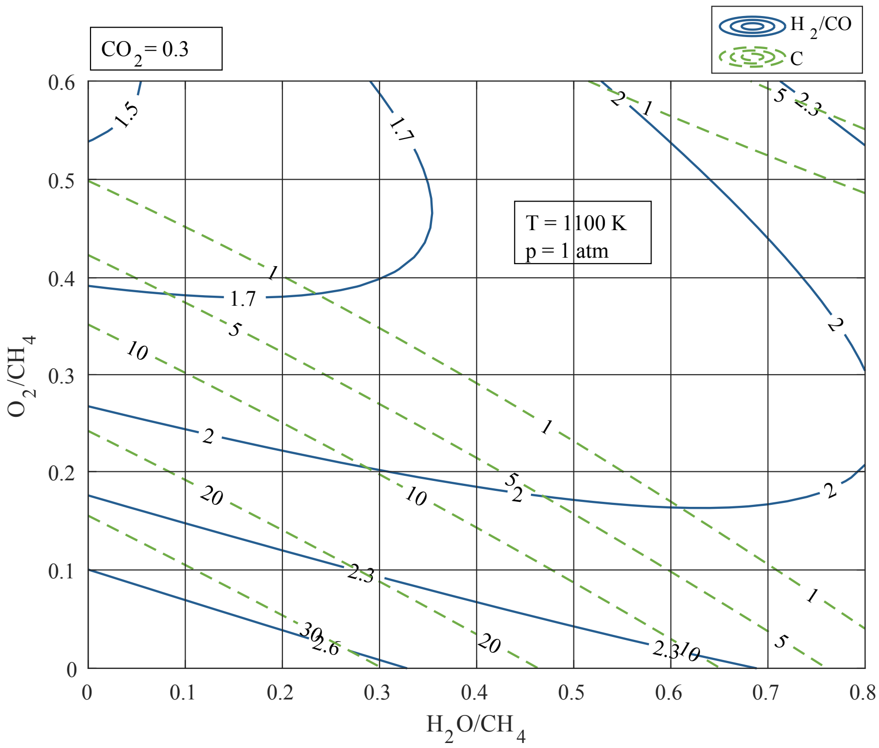

Figure S8. Combined effect of oxygenates (molar H2O/CH4 and O2/CH4 ratio) in the feedstock of TRM process on carbon deposition and H2/CO ratio in syngas; CO2/CH4 ratio=03, T=1100 K, p=1 atm

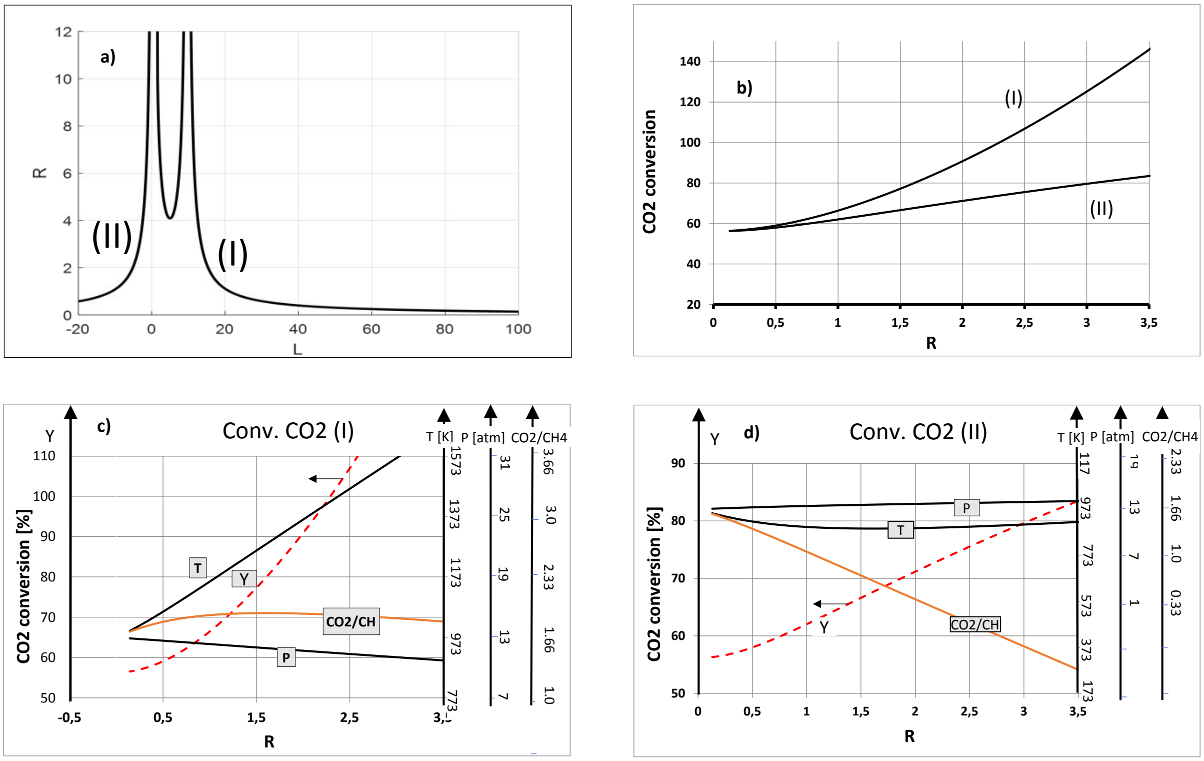

Figure S9. Ridge analysis for conversion CO2 in DRM process: a) Lambda versus R, b) local optimum responses (conversion CO2, case I and II) versus R, c) maximum ridge coordinates for case I, d) maximum ridge coordinates for case II

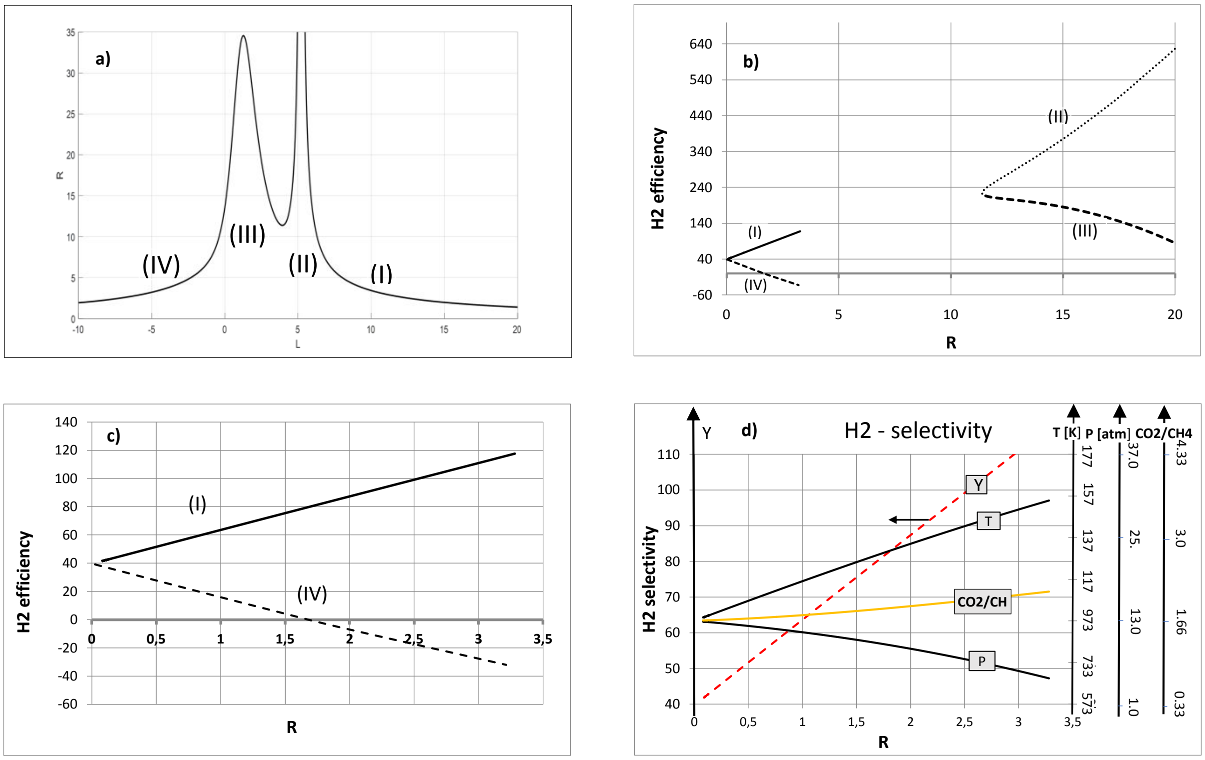

Figure S10. Ridge analysis for H2 efficiency in DRM process : a) Lambda versus R, b) local optimum responses (H2 efficiency, case I - IV) versus R, c) local optimum responses, case I and IV versus R , d) maximum ridge coordinates for case I

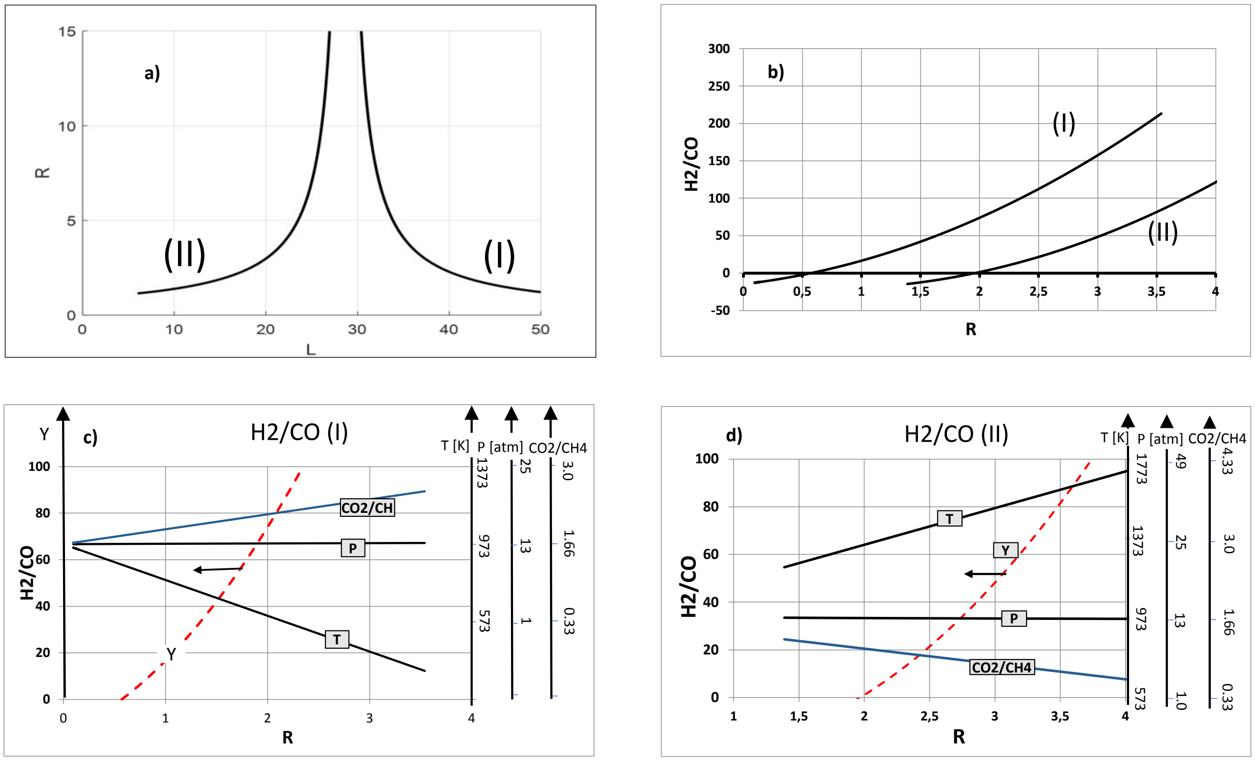

Figure S11. Ridge analysis for H2/CO ratio in syngas for DRM process: a) Lambda versus R, b) local optimum responses (H2/CO ratio, case I and II) versus R, c) maximum ridge coordinates for case I, d) maximum ridge coordinates for case II

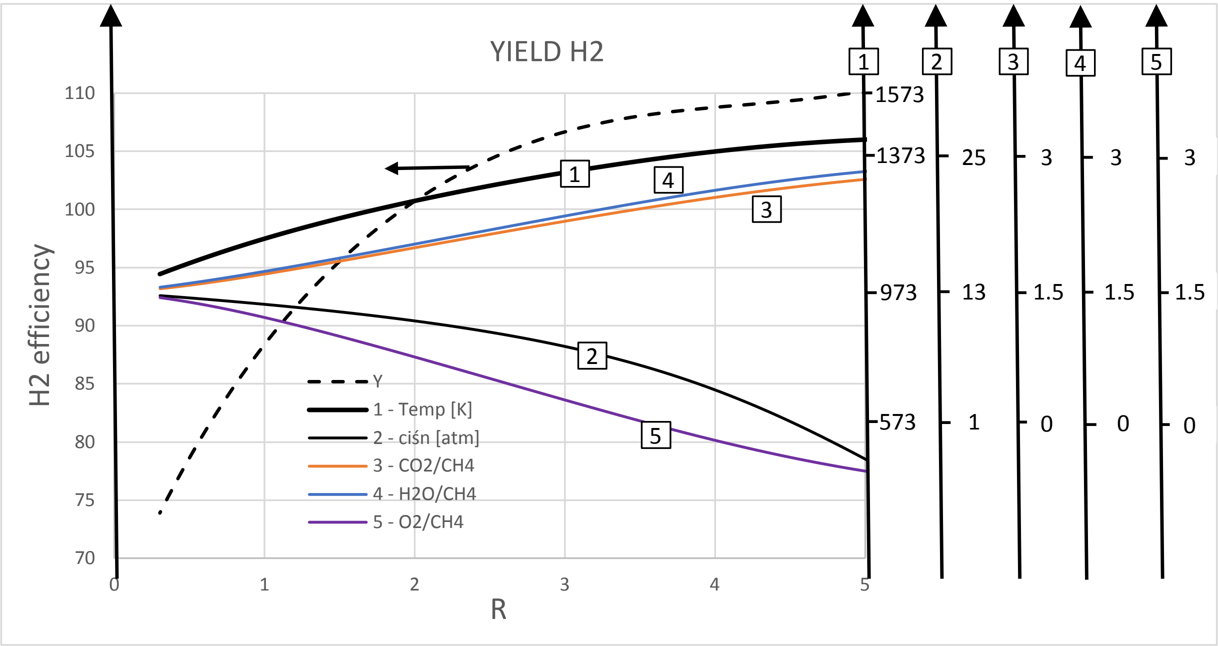

Figure S12. Ridge analysis : optimum ridge coordinates versus R for H2 efficiency in TRM process

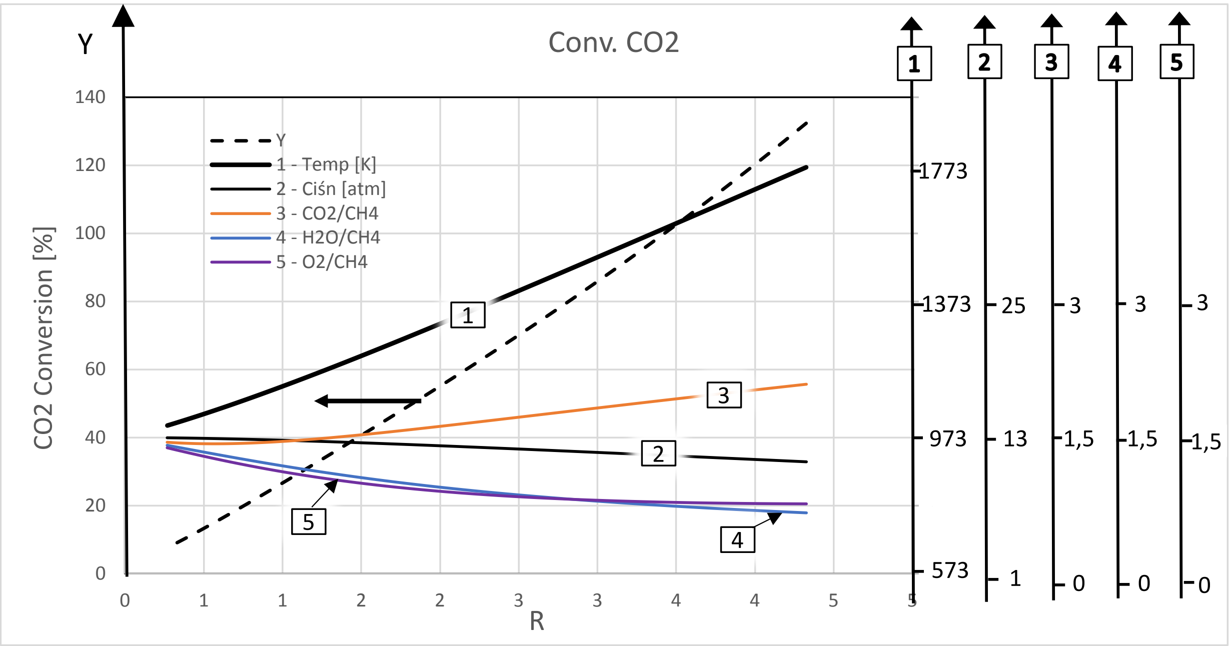

Figure S13. Ridge analysis : optimum ridge coordinates versus R for conversion of CO2 in TRM process
